# A Structure Design Method for Reduction of MRI Acoustic Noise

**DOI:** 10.1155/2017/6253428

**Published:** 2017-11-06

**Authors:** Jiaofen Nan, Nannan Zong, Qiqiang Chen, Liangliang Zhang, Qian Zheng, Yongquan Xia

**Affiliations:** School of Computer and Communication Engineering, Zhengzhou University of Light Industry, Zhengzhou 450000, China

## Abstract

The acoustic problem of the split gradient coil is one challenge in a Magnetic Resonance Imaging and Linear Accelerator (MRI-LINAC) system. In this paper, we aimed to develop a scheme to reduce the acoustic noise of the split gradient coil. First, a split gradient assembly with an asymmetric configuration was designed to avoid vibration in same resonant modes for the two assembly cylinders. Next, the outer ends of the split main magnet were constructed using horn structures, which can distribute the acoustic field away from patient region. Finally, a finite element method (FEM) was used to quantitatively evaluate the effectiveness of the above acoustic noise reduction scheme. Simulation results found that the noise could be maximally reduced by 6.9 dB and 5.6 dB inside and outside the central gap of the split MRI system, respectively, by increasing the length of one gradient assembly cylinder by 20 cm. The optimized horn length was observed to be 55 cm, which could reduce noise by up to 7.4 dB and 5.4 dB inside and outside the central gap, respectively. The proposed design could effectively reduce the acoustic noise without any influence on the application of other noise reduction methods.

## 1. Introduction

Split Magnetic Resonance Imaging (MRI) scanner, as an essential part of a Magnetic Resonance Imaging and Linear Accelerator (MRI-LINAC) system, is used to provide superior images and guide LINAC operation on targeted regions such as tumorous tissue. However, its development has been facing a number of significant challenges. One of the challenges is the acoustic noise from the split gradient coil [1]. Previous studies have reported that the noise in an MRI system may incur hearing loss under exposure to noise without protection [1–3]. Most often, the ways to protect hearing are with earplugs, earmuffs, and helmet during MRI scanning [4]. Damping and sound absorption materials are often applied to the internal structure of an MRI scanner [5]. In addition, there are also reports about active vibration control of gradient coils [6] and pulse alterations [7, 8]. However, the noise is still annoying for a patient after the above processing [9]. Therefore, it is necessary to further attenuate the noise in an MRI system for patients' comfortableness.

Previous studies have reported that asymmetric acoustic design can be tactfully used to achieve noise attenuation. For example, the insertion of an acoustic screen can divide the acoustic path into two asymmetric tunnels, which give rise to a large sound attenuation in a narrow-band content [10]. The specially designed asymmetric inlet and outlet configurations for acoustic silencers could effectively improve the transmission loss [11]. Asymmetric acoustic liners can preferentially enhance sound suppression in a preset direction for sound absorption within an acoustic duct [12]. A circular asymmetric Helmholtz resonator can provide good performance about acoustic offset on the resonant frequency [13]. Therefore, we consider an asymmetric configuration to design the split MRI gradient assembly in this work. Designing two gradient assembly cylinders with different lengths may reduce the simultaneous resonant vibration and counteract the acoustic waves in the cylindrical tunnels at the critical frequency band, which may reduce the noise level.

In addition, it has been reported that the horn structure can be used to transmit sound waves and abate reflection at the open ends in the MR gradient coils [14]. The horn structure acts as a waveguide by making continuous impedance difference from the gradient assembly bore to the outside free space. A well-designed acoustic horn can efficiently transmit the incoming wave energy and favourably distribute it to the far field space [15–19]. Similarly, the horn structure can be applied to the outer ends of the split MRI scanner and transmit the sound energy from the cylindrical tunnels to the outside space. Therefore, we also designed the split main magnet ends with horn structure for further noise reduction.

The current design can adjust the vibration response of MRI-LINAC system by asymmetric gradient assembly and play a role of waveguide to transmit the acoustic waves outside the cylindrical tunnels by the horn structure. The performance on noise reduction will be assessed quantitatively using the finite element method (FEM). In the study, a two-dimensional (2D) axisymmetric FE model of a split MRI system was built to evaluate the effectiveness of the noise reduction based on the design of the asymmetric gradient assembly and horn structures on the outer ends of the split magnet.

## 2. Methods

### 2.1. A 2D FE Acoustic Model for a Split MRI Scanner

**Figure 1:**
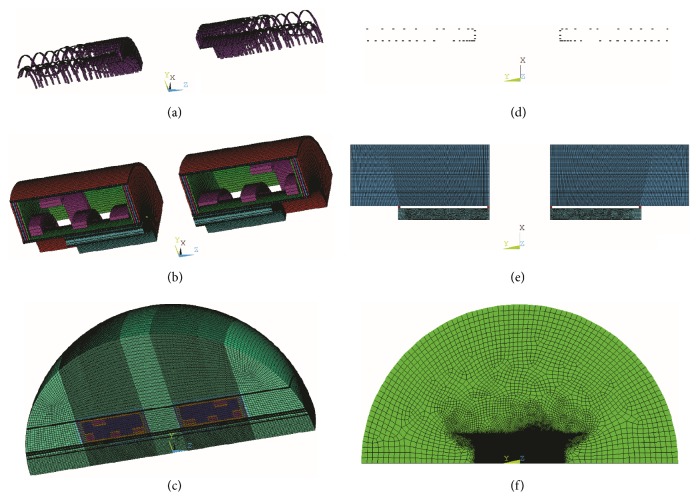
Acoustic FE model of the split MRI system: (a–c) 3D 1/4 model; (d–f) 2D axisymmetric model. (a) Split gradient coils including the *x*, *y*, and *z* coils, (b) split MRI system, (c) 3D acoustic model of the split MRI system including the surrounding air, (d) split gradient coils including the *z* coils, (e) simplified split MRI system, and (f) 2D acoustic model of the split MRI system including the surrounding air.

**Figure 2:**
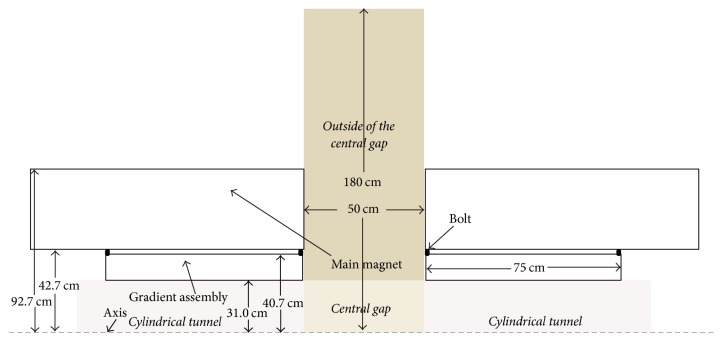
Dimensions and regional divisions of the split MRI system (2D axisymmetric diagram). The central gap investigated here is the area between the two cylindrical tunnels, and the area outside the central gap is from the axis of the split gradient assembly outwards to 180 cm, which approximates to the height of an adult.

Since the gradient coils have little influence on the whole vibration mode of the gradient assembly [20], we simulated only the *z* coils in the gradient assembly in our finite element modeling work for computational simplification. The model setup is illustrated in Figure 1. Gradient pulses with sinusoidal waveforms were applied with peak value of 600 A (producing a gradient strength of 20 mT/m in the imaging area) and the magnetic flux density of the main magnet is 1 T. The commercial program ANSYS was used for the above simulations. We note that the 3D FE model of the whole gradient assembly (see Figures 1(a)–1(c)) was set up for the full investigation of the split MRI system [1, 21]. However, since 3D model has much larger DOF (degrees of freedom) than 2D model, its computational cost is much higher than that of 2D model. Moreover, switching from 3D to 2D model has little effect on the precision of the estimated dampening of noise for the *z* coils. Therefore, a 2D FE modeling work was conducted for the simulation of only *z* coils (see Figures 1(d)–1(f)).  Figure 2 illustrates the dimensions and the regional divisions of the split MRI system. For brain imaging/treatment, the patient's ears will reside in the central gap. However, if the system is used to image/treat the torso, pelvis, or extremities, the patient's ears will be positioned outside the central gap. Therefore, the investigation of the acoustic control here focuses on both the inside and the outside of the central gap.

### 2.2. Asymmetric Design of the Split Gradient Assembly

In the designed MRI-LINAC system, the patient bed is installed perpendicular to the axis of the main magnet, where the total acoustic field is the superposition of the acoustic fields generated from those two vibrating gradient assembly cylinders. For two identical gradient assembly cylinders of a split MRI system, they have the same resonant frequencies and vibration modes. During the operation of the MRI scanner, the two cylinders are energized simultaneously with symmetric or antisymmetric Lorentz forces, producing equal-amplitude acoustic waves. The superposition of two intense acoustic fields will produce high SPL (sound pressure level) in the split MRI system. However, if the two cylinders are different in length, they will have distinct set of vibration modes. The acoustic waves produced by the two gradient assembly cylinders will have different amplitudes at the same frequency. This will avoid the superposition of two strong acoustic waves. In addition, the asymmetric design of the split gradient assembly may result in the fact that the acoustic waves radiated from the two cylinders have more than a 90-degree phase difference at some frequencies, thus producing a mutual counteraction of the acoustic waves. Therefore, this asymmetric structure will smooth the acoustic response spectrum of the split gradient assembly and produce a relatively uniform acoustic response with low amplitudes at resonant frequencies.

**Figure 3:**
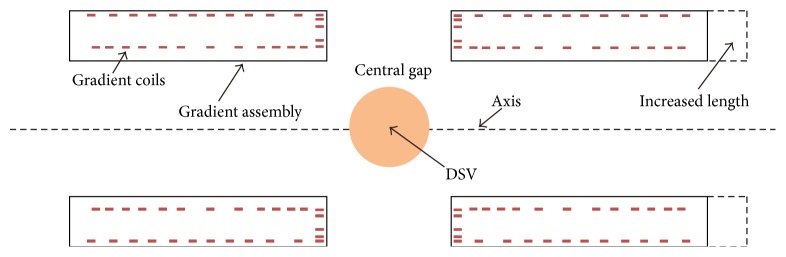
Diagram of the asymmetric design of the split gradient assembly.

In this work, the asymmetric gradient assembly was designed by an increase of right side of the gradient assembly cylinder, as is shown in Figure 3 (in Figure 2, the shown gradient assembly length is initial, which is a little longer than the gradient coils). The acoustic responses of the split gradient assembly were investigated to identify the resonant frequencies. Different cases were explored with increased lengths from 1 cm to 25 cm with 1 cm interval and their acoustic responses were compared with each other to find the most proper asymmetric design.

### 2.3. The Design of a Horn-Shaped Structure for the Ends of the Split Main Magnet

**Figure 4:**
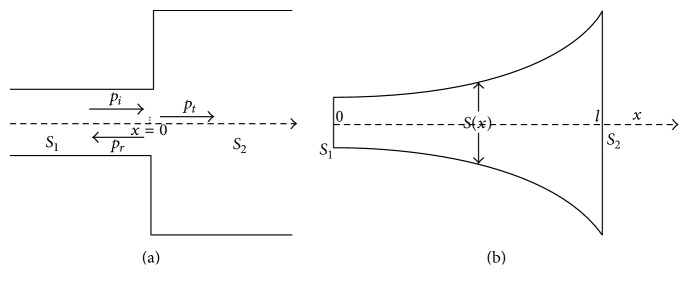
Acoustic wave propagation in the pipes: (a) acoustic wave propagation in a rigid pipe with two variational cross sections and (b) acoustic wave propagation in a rigid horn.

In a tube with a varying cross section as is shown in Figure 4(a), due to the change of the acoustic impedance, some acoustic waves will reflect at the variational interface and others will transmit through. However, if the cross section gradually varies from a smaller area to a larger one, for example, like a horn as is shown in Figure 4(b), the acoustic impedance variation will be continuous. Thus, most acoustic energy will be transmitted from the left (the horn mouth) to the right open space, so the reflective acoustic waves will be much less.

**Figure 5:**
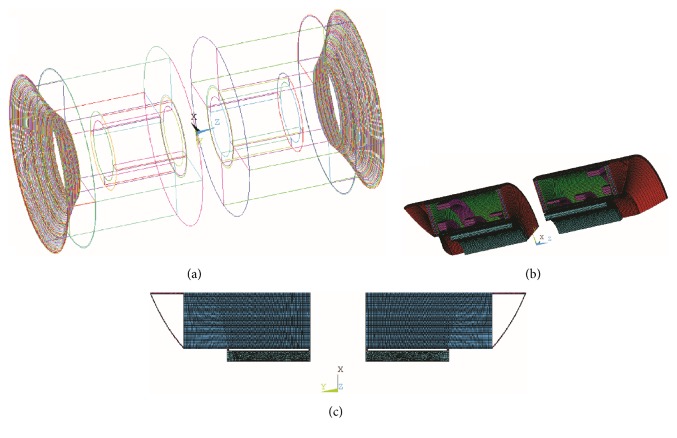
Acoustic horn installation way on the split MRI system: (a) 3D line structure of the split MRI system with acoustic horns, (b) 3D meshed split MRI system with acoustic horns (1/4 model), and (c) 2D meshed split MRI system with acoustic horns.

In this work, two acoustic horns were installed symmetrically on the outer ends of the main magnet of the split MRI system after the asymmetric gradient assembly design.  Figure 5 shows the acoustic horn installation way on the split MRI system. The cross section areas of the horn structures are obtained using A.6 (see Appendix). In the simulation, the length of the horn *l* was altered from 10 cm to 100 cm with 5 cm interval in order to find an optimal configuration for the purpose of noise reduction inside and outside the central gap. The mechanical properties of the scanner components and the designed horn were displayed in Table 1.

## 3. Results

### 3.1. Acoustic Performance Evaluation of the Asymmetric Split Gradient Assembly

Simulation shows that the SPLs for both the 3D model and the simplified 2D model range from around 60 dB to around 140 dB. The acoustic responses of the 2D model have consistent trend and approximate amplitude to the 3D model. After the comparison, the 2D model was used to investigate the proposed noise reduction scheme.

**Figure 6:**
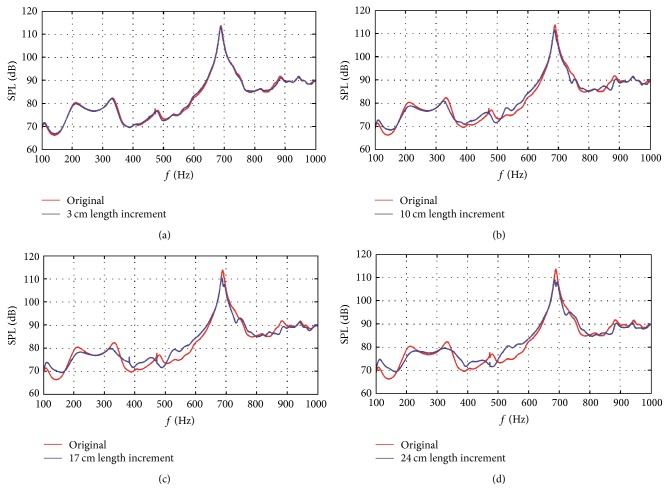
Acoustic response comparisons between the original symmetric gradient assembly and the asymmetric design with (a) 3 cm, (b) 10 cm, (c) 17 cm, and (d) 24 cm length increments.

Figure 6 shows the acoustic responses at different frequencies of four asymmetric gradient assembly designs from 100 Hz to 1000 Hz. From Figure 6, there are no severe fluctuations of acoustic responses in this frequency extent. However, there exist some frequency bands that have local SPL extremums such as 200–240 Hz, 305–345 Hz, and 660–720 Hz. By designing the split gradient assembly asymmetrically, the SPLs at these frequency bands were effectively attenuated. However, acoustic responses are increased at some frequency bands. In fact, most of the frequency bands with increased SPL are nonresonant vibration bands that have little contribution to the total SPL of the MRI sequence. Usually, the SPL at the resonant frequency bands is much higher than that at the nonresonant vibration bands, so the resonant frequency bands play a decisive role in the total SPL. For the noise reduction effect, interest here was focused on frequency bands with local SPL extremums. The maximum SPL reductions in these frequency bands after employing the asymmetric design were then calculated for different length increments, from 1 cm to 25 cm with 1 cm interval.

**Figure 7:**
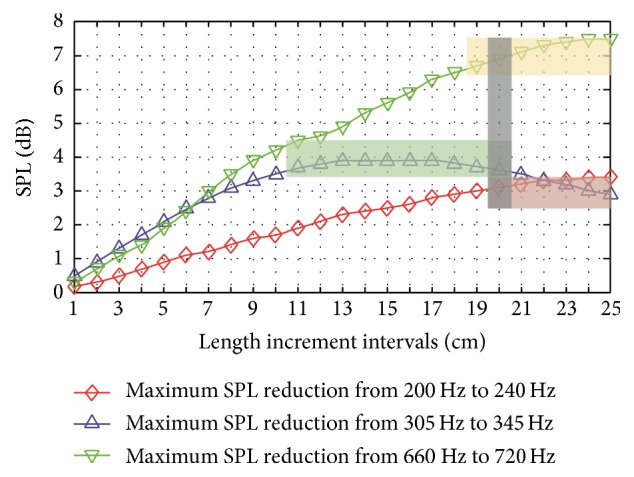
Maximum SPL reductions of the frequency bands of interest in the central gap by using asymmetric gradient assembly designs. The optimal length increment intervals are illustrated using different-colour strips.

Figure 7 shows the maximum SPL reductions of the frequency bands of interest in the central gap by using asymmetric gradient assembly designs. For the frequency band of 200–240 Hz, the maximum SPL reduction in the central gap steadily increases with respect to the length increment. However, for the frequency band from 305 to 345 Hz, the maximum SPL reduction ascends first when the length increment interval is 1–13 cm, and then it remains constant at the maximum value among the interval of 13–17 cm, and, afterwards, it goes down with respect to the increment from 17 cm to 25 cm because of the reduction in the effect of the acoustic wave phases difference. When the length increment is from 1 cm to 21 cm, the maximum SPL reduction at the frequency band of 305–345 Hz is larger than the maximum SPL reduction at the frequency band of 200–240 Hz, but it becomes smaller when the increment is from 23 cm to 25 cm. Comparatively, the maximum SPL reduction for the frequency band of 660–720 Hz has a similar variation trend to the maximum SPL reduction for the frequency band of 200–240 Hz, but the former has larger SPL reductions than the latter. Here, for the three frequency bands, the selected criteria of the maximum SPL reduction are shown in Table 2. According to the criteria in Table 2, the optimal length increment intervals of the maximum SPL reduction for the frequency bands of 200–240 Hz, 305–345 Hz, and 660–720 Hz are 20–25 cm, 11–20 cm, and 19–25 cm, respectively. The overlapped optimal length increment quantity of the maximum SPL reduction for the three criteria is 20 cm. The optimal length increment intervals are illustrated in Figure 7 using different-colour strips.

**Figure 8:**
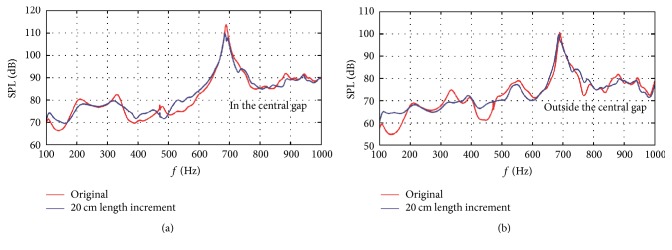
Acoustic response comparisons between the original symmetric gradient assembly and the asymmetric design with 20 cm length increment: (a) acoustic response comparison inside the central gap and (b) acoustic response comparison outside the central gap.

Figure 8 shows the acoustic response comparisons between the original symmetric gradient assembly and the asymmetric design with 20 cm length increment. From Figure 8(a), it can be seen that the frequency bands with local SPL extremums are obviously attenuated in the central gap. When the split gradient assembly was designed asymmetrically, the original SPL peak at around 689 Hz was divided into two low-amplitude peaks, which indicate the respective resonant peaks of the two gradient cylinders. For the outside of the central gap in Figure 8(b), there are slight SPL reductions at the frequency bands of 200–240 Hz and 660–720 Hz. However, the SPL reduction is still obvious at frequency band of 305–345 Hz. There are also other frequency bands where the SPL peaks are attenuated such as 500–600 Hz and 850–900 Hz.

**Figure 9:**
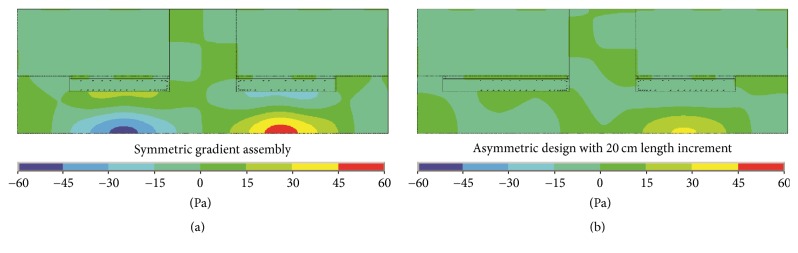
Acoustic field distribution in the split MRI system with single-frequency gradient pulse input at 689 Hz: (a) acoustic field distribution of the symmetric gradient assembly and (b) acoustic field distribution of the asymmetric design with 20 cm length increment.

Table 3 shows the summarized maximum SPL reductions both inside and outside the central gap at the frequency bands of interest by applying an asymmetric gradient assembly design with the optimal 20 cm length increment. From (2) (see Discussion), it can be deduced that there is acoustic-wave offset effect in the central gap at the frequency band of 660–720 Hz, where the maximum SPL reduction amounts to 6.9 dB (larger than 6.0 dB).  Figure 9 shows the acoustic field distribution in the split MRI system with single-frequency gradient pulse input at 689 Hz. The acoustic-field offset effect can be observed from a comparison between Figures 9(a) and 9(b). For the asymmetric design, the acoustic pressure intensity is weaker than the symmetric gradient assembly, especially in the cylindrical tunnels. At this frequency, the original gradient cylinder is resonant but the length-increased one is not, which makes the whole acoustic field attenuated. Overall, the asymmetric design of the split gradient assembly can reduce the acoustic responses at frequency bands with local SPL extremums and thus smooth the acoustic responses.

### 3.2. Acoustic Performance Evaluation of the Horn Structures

In order to smoothly transmit the intense acoustic waves out of the cylindrical tunnels, horn structures were designed on the outer ends of the split main magnet after the optimal asymmetric gradient assembly design. The acoustic effect was also focused on the frequency band from 100 Hz to 1000 Hz.

**Figure 10:**
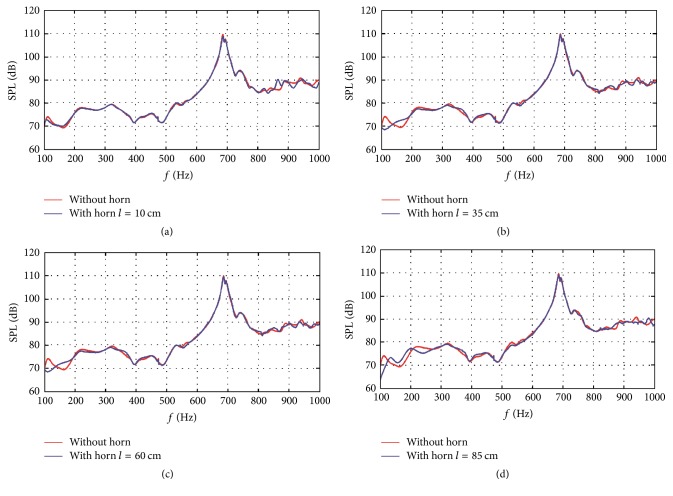
Acoustic response comparisons between structures with and without horn. The horn lengths are (a) 10 cm, (b) 35 cm, (c) 60 cm, and (d) 85 cm. For the case without horn, the acoustic response is that of the asymmetric gradient assembly configuration with 20 cm length difference.

Figure 10 shows the acoustic responses at different frequencies of four horn designs. The acoustic response marking without horn structure is that of the asymmetric gradient assembly design at the optimal length increment of 20 cm. From Figure 10, by using horn structure, the frequency band of 100–140 Hz is obviously attenuated and the selected three frequency bands in the asymmetric gradient assembly design section are further attenuated, although SPL reductions are slight. Similar to the asymmetric gradient assembly design, interest here was focused on these four frequency bands. The maximum SPL reductions in these frequency bands after applying horn structures were calculated with respect to different horn lengths from 5 cm to 100 cm with 5 cm interval.

**Figure 11:**
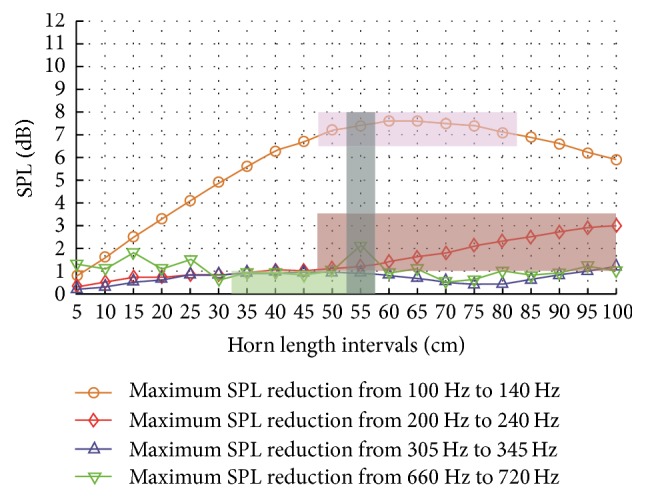
Maximum SPL reductions of the frequency bands of interest in the central gap by using horn structures. The optimal horn length intervals are illustrated using different-colour strips. The SPL reductions were compared between structures with and without horn after applying the optimal asymmetric gradient assembly design.

**Figure 12:**
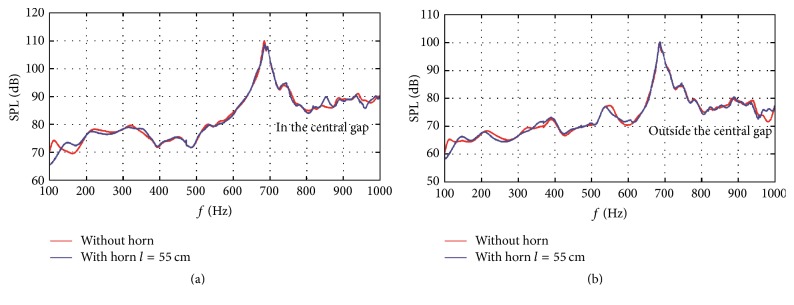
Acoustic response comparisons between structures without horn and with horn at the optimal 55 cm length: (a) acoustic response comparison inside the central gap and (b) acoustic response comparison outside the central gap.

Figure 11 shows the maximum SPL reductions of the frequency bands of interest in the central gap by using horn structures. For the frequency band of 100–140 Hz, the maximum SPL reduction goes up when the horn length is increased from 5 cm to 60 cm, but then it descends slightly when the horn length is larger than 65 cm because of a decrease in the match of the configuration of the horn and the acoustic wave length. However, for the frequency bands of 200–240 Hz, 305–345 Hz, and 660–720 Hz, the maximum SPL reductions fluctuate between 0.2 dB and 3.0 dB, of which the scale is much smaller than that at the frequency band of 100–140 Hz. Here, for the four frequency bands of interest, the selected criteria of the maximum SPL reduction are shown in Table 4. According to the four criteria in Table 4, the optimal horn length intervals of the maximum SPL reduction for the frequency bands of 100–140 Hz, 200–240 Hz, 305–345 Hz, and 660–720 Hz are 50–80 cm, 50–100 cm, 35–55 cm, and 55 cm, respectively. The overlapped optimal horn length quantity of the maximum SPL reduction for the four criteria is 55 cm. The optimal horn length intervals are illustrated in Figure 12 using different-colour strips.

Figure 12 shows the acoustic response comparisons between structures without horn and with horn at the optimal length of 55 cm. From Figure 12(a), it can be seen that the frequency bands of interest with local SPL extremums are further attenuated in the central gap after applying horn structure. The SPL peak at the frequency band of 660–720 Hz is further reduced by 2.1 dB. For region outside of the central gap in Figure 12(b), although the SPL reduction effect is not as good as that in the central gap, it still has 5.4 dB reduction at the frequency band of 100–140 Hz. Another frequency band of 530–570 Hz which has local SPL extremum is also slightly attenuated.

Table 5 shows the summarized maximum SPL reductions both inside and outside the central gap at the frequency bands of interest by applying horn structure with the optimal 55 cm length. After applying the horn structure, the SPL at the frequency band of 100–140 Hz is obviously reduced.

**Figure 13:**
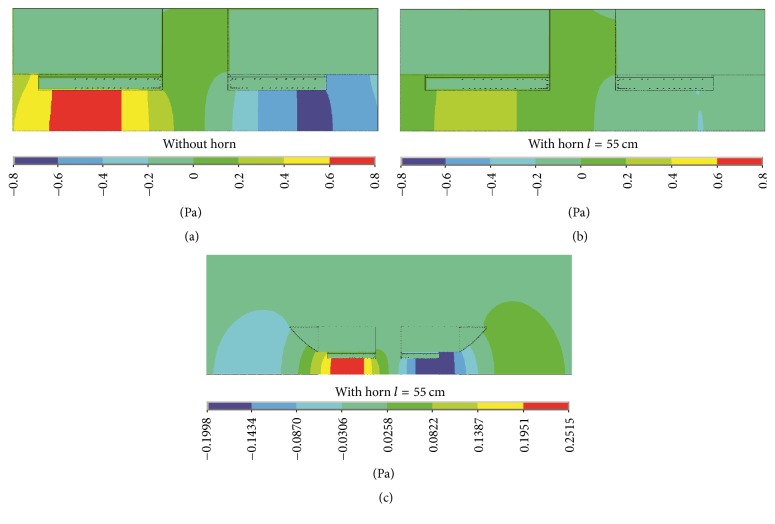
Acoustic field distribution with single-frequency gradient pulse input at 110 Hz: (a) acoustic field distribution in the split MRI system without horn, (b) acoustic field distribution in the split MRI system with horn of 55 cm length, and (c) acoustic field distribution in and around the split MRI system with horn of 55 cm length. For (c), it has the same result with different legend scale from (b) so as to clearly display the wave guide effect by applying horn structure.

Figure 13 shows the acoustic field distribution in and around the split MRI system with single-frequency gradient pulse input at 110 Hz. Comparing Figures 13(a) and 13(b), it can be seen that the acoustic field intensity in the split MRI system is largely attenuated by applying the horn structure, especially in the central gap and the cylindrical tunnels. From Figure 13(c), the horns behave like wave guide structures which effectively transmit the intense acoustic field outside the MRI system.

## 4. Discussion

When investigating the proposed noise reduction scheme, two simulation problems were found, which can potentially impact the solution quality. The first one is the material damping properties and the other one is the simulation errors at high-frequency band. For the first problem, a small damping factor was added on the epoxy resin simulation to avoid extreme sound pressure. Theoretically, from (1), if there is no damping coefficient *c*, the vibration amplitude can be infinite, which is not the real situation. For the second problem, assume that there is a single DOF system, of which the resonant frequency is as (1), where *F*_*A*_ is the amplitude of a sinusoidal exciting force, *ω* is the angular frequency, *c* is the viscous damping coefficient, *M* is the mass, and *K* is the elastic stiffness. If there are two resonant frequencies *ω*_1_ and *ω*_2_ which are very close, very small frequency resolution will be needed to distinguish them. However, for the FE method, the mesh cannot be infinitely small, which may produce simulation errors at the mode-dense frequency band if the mesh generation is changed (when increasing the length of one gradient cylinder, the FE mesh will be slightly different from the original). For the asymmetric design, the superposed SPL is as (2), where *p*_1_ and *p*_2_ are the amplitudes of two incident acoustic waves, *θ* is their phase difference, and *p*_0_ is the referential pressure. Normally, if one gradient cylinder is resonant but the other one is not, then *p*_1_ ≪ *p*_2_ or *p*_2_ ≪ *p*_1_; the SPL can be decreased by about 6 dB compared with the situation when the two gradient cylinders are simultaneously resonant. When considering the acoustic-wave offset effect, the SPL difference should be around 6 dB (slightly larger or smaller). Comparatively, at the low-frequency bands, the mode density is sparse and the acoustic wave lengths are large. The simulation accuracy can be guaranteed even with large-size meshes. Besides, the energies of most of the gradient pulses concentrate at low-frequency band, such as GE (gradient echo) and SE (spin echo) [7, 22, 23]. Therefore, the investigation in this work focuses on the frequency band from 100 Hz to 1000 Hz, which can present a more clear observation of the noise reduction scheme effect and the method may still suit the high-frequency condition.

(1) ξA=FAωc2+ωM−K/ω2

(2) SPL=10log⁡p12+p22+2p1p2cos⁡θ2p02.

The noise reduction scheme proposed in this work does not affect the application of traditional noise control processing, such as damping materials or sound absorption materials application. With all the noise reduction methods combined together, the overall noise reduction effect will be much considerable.

## 5. Conclusions

This work numerically investigated two noise reduction methods for a split gradient coil in an MRI-LINAC system. The length difference between the two gradient coil cylinders resulted in an effective SPL reduction inside and outside of the central gap. At frequency bands of 200–240 Hz, 305–345 Hz, and 660–720 Hz, the SPL extremums were reduced by applying asymmetric gradient assembly. At the optimal length increment of 20 cm, the SPL of a dominant resonant frequency was attenuated by 6.9 dB in the central gap and also achieved a 5.6 dB SPL reduction outside the central gap at a frequency band with local SPL extremum, which eventually smoothed the acoustic response spectrum. Using an additional method, the horn structures were designed on the ends of the split main magnet. This processing successfully transmitted the acoustic waves outside of the cylindrical tunnels of the split gradient assembly, thus reducing the noise level inside the scanner. The FE simulation suggested that, with an optimal horn length of 55 cm, the SPL values at typical frequencies could be dropped by 7.4 dB inside the central gap and by 5.4 dB outside the central gap. Overall, by using asymmetric gradient assembly design and applying horn structure, the maximum SPL reduction at a dominant resonant frequency band of 660–720 Hz amounted to 9.0 dB. Future experimental validation will be performed to verify the proposed noise reduction scheme.

## Figures and Tables

**Table 1:** Mechanical properties of the acoustic model components.

Item	*E* (GPa)	*μ*	*ρ* (kg/m^3^)	*c* (m/s)
Gradient assembly	20	0.30	3000	
Gradient *z* coils	117	0.34	8960	
Bolts between the gradient assembly and main magnet	210	0.3	7800	
Main magnet	210	0.3	7800	
Horn structure	40	0.3	2550	
Surrounding air			1.225	340

*E*, *μ*, *ρ*, and *c* are Young's modulus, Poisson's ratio, density, and sound velocity, respectively. Since *x* and *y* coils were not simulated in the 2D model, Yong's modulus and density of the gradient assembly were set to be a little larger compared with our previous 3D model. The main magnet was simulated as two steel cylinders in the 2D model.

**Table 2:** Maximum SPL reductions of the frequency bands of interest in the central gap by applying asymmetric gradient coil assembly designs.

Frequency bands of interest	200–240 Hz	305–345 Hz	660–720 Hz
Selected criteria (dB)	3.0	3.5	6.5
Optimal length increment interval (cm)	20–25	11–20	19–25

**Table 3:** Maximum SPL reductions both in and outside the central gap at the frequency bands of interest by applying asymmetric gradient assembly design with the optimal length increment of 20 cm.

Frequency band	200–240 Hz	305–345 Hz	660–720 Hz
In the central gap	3.1	3.6	6.9
Outside the central gap	0.9	5.6	3.5

**Table 4:** Maximum SPL reductions of the frequency bands of interest in the central gap by applying horn structures.

Frequency bands of interest	100–140 Hz	200–240 Hz	305–345 Hz	660–720 Hz
Selected criteria (dB)	7.0	1.0	0.8	2.0
Optimal horn length (cm)	50–80	50–100	35–55	55

**Table 5:** Maximum SPL reductions both in and outside the central gap at the frequency bands of interest by applying the horn structure with an optimal length of 55 cm.

Frequency band	100–140 Hz	200–240 Hz	305–345 Hz	660–720 Hz
In the central gap	7.4	1.2	0.9	2.1
Outside the central gap	5.4	1.6	1.2	0.6

## Appendix

Assume that there is a rigid pipe with two variational cross sections, of which the acoustic impedance changes from *Z*_1_ to *Z*_2_, as is shown in Figure 4(a). Due to the change of the acoustic impedance, some acoustic waves will reflect at the variational interface and others transmit through. In Figure 4(a), the incident wave is *p*_*i*_, the reflective wave is *p*_*r*_, the transmissive wave is *p*_*t*_, the cross section area of the incident pipe is *S*_1_, the cross section area of the transmissive pipe is *S*_2_, and the pipe length is infinite. The acoustic pressures can be expressed as [19]

(A.1) pi=paiejωt−kxpr=parejωt+kxpt=patejωt−kx

and the air particle velocities are [19]

(A.2) vi=paiρ0c0ejωt−kxvr=−parρ0c0ejωt+kxvt=patρ0c0ejωt−kx,

where *p*_*ai*_ is the incident acoustic wave amplitude, *p*_*ar*_ is the reflective acoustic wave amplitude, *p*_*at*_ is the transmissive acoustic wave amplitude, *v*_*i*_, *v*_*r*_, and *v*_*t*_ are the corresponding air particle velocities, *ρ*_0_ is the air density, *c*_0_ is the acoustic velocity in the air, *ω* is the angular frequency, and *k* is the wave number defined as *ω*/*c*_0_. *x* = 0 is the variational interface of the pipe cross section, where the following boundary conditions exist [24, 25]:

(A.3) pai+par=patS1vi+vr=S1vt.

The reflective coefficient can be acquired [24, 25]:

(A.4) $$\begin{array}{c} r_{p}=\frac{p_{ar}}{p_{ai}}=\frac{S_{1}/S_{2}-1}{S_{1}/S_{2}+1}. \end{array}$$

If *S*_2_ ≫ *S*_1_, then *r*_*p*_ ≈ −1. The acoustic waves nearly totally reflect back at the interface.

However, if using a horn to transmit the acoustic waves from *S*_1_ to *S*_2_, as shown in Figure 4(b), the acoustic transmission equation will be [26]

(A.5) $$\begin{array}{c} \frac{\partial^{2}p}{\partial x^{2}}+\left(\;\frac{\partial \ln S\left(x\right)}{\partial x}\;\right)\frac{\partial p}{\partial x}=\frac{1}{c_{0}^{2}}\frac{\partial^{2}p}{\partial t^{2}}. \end{array}$$

If the cross section area of the horn obeys to the principle

(A.6) $$\begin{array}{c} S\left(\;x\;\right)=S_{1}e^{\delta x}, \end{array}$$

the acoustic pressures can be acquired:

(A.7) p=paie−δ/2x+jωt−k2−δ2/4x+pare−δ/2x+jωt+k2−δ2/4x,

where *δ* is the winding index.

If the acoustic impedance at the sound propagation end of the horn is *Z*, the reflective coefficient can be solved [24, 25]:

(A.8) rp=parpai=e−2jlk2−δ2/4Zejθ−ρ0c0/S2Zejθ+ρ0c0/S2θ=tan−1⁡δ2k2−δ2/4,

where *l* is the length of the horn. If the radius of the sound propagation end of the horn is large enough, the acoustic impedance there can be approximated to be *Z* = *ρ*_0_ *c*_0_/*S*_2_. Thus, |*r*_*p*_| < 1; the reflective acoustic energy will be attenuated.
